# Silver Nanoparticles Synthesized From *Centella asiatica* Extract and Asiatic Acid for Enhanced Biofilm Eradication of *Streptococcus* Associated With Oral Diseases

**DOI:** 10.1155/sci5/4867529

**Published:** 2025-04-15

**Authors:** Sukanlaya Leejae, Wattana Pelyuntha, Lavanya Goodla, Auemphon Mordmuang

**Affiliations:** ^1^School of Languages and General Education, Drug and Cosmetics Excellence Center, Walailak University, Nakhon Si Thammarat 80160, Thailand; ^2^Futuristic Science Research Center, School of Science, Walailak University, Nakhon Si Thammarat 80160, Thailand; ^3^Department of Biochemistry and Molecular Biology, MSC08 46701 University of New Mexico, Albuquerque, New Mexico 87131-0001, USA; ^4^Department of Medical Sciences, School of Medicine, Walailak University, Nakhon Si Thammarat 80160, Thailand

**Keywords:** biofilm, *Centella asiatica*, cytotoxicity, oral diseases, silver nanoparticles, *Streptococcus*

## Abstract

The biofilm-forming ability of *Streptococcus* species, particularly *Streptococcus mutans*, is a key factor in the pathogenesis of dental caries and periodontitis. Current treatments often exhibit limitations such as incomplete biofilm eradication and cytotoxicity to host tissues, highlighting the need for innovative and biocompatible therapeutic approaches. Therefore, this study aimed to investigate the potential of *Centella asiatica* ethanolic extract, its bioactive triterpenoids (asiatic acid and madecassic acid), and silver nanoparticles (AgNPs) synthesized from the extract as an alternative strategy for targeting *S. mutans* biofilms. The antibacterial and antibiofilm activities of the synthesized AgNPs against *Streptococcus* species were evaluated, alongside cytotoxicity assessments on human gingival fibroblast (HGF-1) cells using the MTT assay. The synthesized AgNPs exhibited superior antimicrobial efficacy compared to the extract, with significantly lower minimum inhibitory concentration (MIC) and minimum bactericidal concentration (MBC) values (62.5/125 µg/mL) against *S. mutans* ATCC 25175 and *S. mitis* ATCC 49456, highlighting their potent bactericidal activity. Moreover, the AgNPs achieved rapid biofilm disruption, reducing biofilm biomass by 76% within 12 h at 1/2 × MIC, significantly outperforming the extract and triterpenoids. Scanning electron microscopy further revealed substantial extracellular polymeric substance degradation and biofilm structural disruption upon AgNP treatment, confirming their pronounced antibiofilm efficacy. In addition, the synthesized AgNPs demonstrated favorable biocompatibility, maintaining 68% cell viability in dental fibroblast cells, suggesting an optimal balance between antimicrobial potency and cytotoxicity. The synergistic interaction between AgNPs and *C. asiatica* phytochemicals significantly enhanced biofilm disruption compared to nonfunctionalized AgNPs. These findings underscore the potential of *C. asiatica*–based AgNPs as a novel, plant-derived nanotechnological approach for managing oral infections caused by biofilm-forming *Streptococcus* species. This study not only contributes to the development of alternative antibiofilm strategies but also paves the way for future clinical applications in oral healthcare.

## 1. Introduction

Oral diseases, including dental caries and periodontitis, affect over 3.5 billion people globally and represent a significant public health challenge, particularly in low- and middle-income countries where access to dental care is limited [1]. *Streptococcus mutans*, *S. mitis*, and *S. salivarius* are key contributors to these conditions through biofilm formation. These structured microbial communities, encased in an extracellular polymeric substance (EPS), protect bacteria from environmental stressors and antimicrobial agents, thereby facilitating adhesion and pathogenicity [2]. The acidogenic properties of *S. mutans* accelerate enamel demineralization, initiating dental caries, while persistent biofilms exacerbate periodontal inflammation, increasing systemic health risks such as cardiovascular diseases and diabetes [3, 4]. Moreover, the increasing prevalence of antimicrobial resistance among oral streptococci has further complicated biofilm management, reducing the efficacy of conventional antibiotics and antiseptics [5, 6]. Multidrug-resistant *S. mutans* strains have been reported in clinical settings, necessitating novel therapeutic approaches that target biofilm-specific mechanisms [7].

The primary strategies for managing oral biofilms involve chemical agents such as chlorhexidine, fluoride, and antibiotics. Although effective in reducing microbial loads, these agents have significant drawbacks, including mucosal irritation, tooth staining, dysbiosis, and the emergence of antibiotic resistance [8]. These challenges highlight the critical need for alternative, safer, and biocompatible therapeutic options. In recent years, natural products have gained attention as sustainable alternatives for biofilm management. Research findings have shown that natural bioactive compounds from medicinal plants can interfere with quorum sensing, a bacterial communication system that regulates biofilm development and virulence factor production [9–11]. *Centella asiatica*, a medicinal herb long used in wound healing, has demonstrated significant potential against bacterial biofilms and oral pathogens. Asiatic acid and madecassic acid, two prominent triterpenoids isolated from *C. asiatica*, exhibit diverse pharmaceutical activities relevant to biofilm-associated oral diseases [12]. Asiatic acid disrupts biofilm formation by inhibiting glucosyltransferases (Gtfs) activity, reducing EPS matrix synthesis, and targeting bacterial membranes, particularly those of *S. mutans* [13]. Moreover, it exerts anti-inflammatory and antioxidant effects, protecting oral tissues from oxidative stress [14, 15]. Similarly, madecassic acid impairs bacterial adhesion, quorum sensing, and biofilm integrity while promoting fibroblast differentiation and collagen synthesis, enhancing tissue regeneration [16]. Both compounds exhibit selective antimicrobial activity, minimizing harm to beneficial oral microbiota and reducing cytotoxicity risks [17].

Nanotechnology offers innovative solutions for enhancing the efficacy of natural antimicrobial agents. Silver nanoparticles (AgNPs) synthesized using plant extracts demonstrate significant antibiofilm activity [18]. Unlike conventional agents, AgNPs can penetrate EPS matrix, destabilize microbial membranes, and disrupt metabolic pathways, enhancing treatment susceptibility [19]. Previous research has indicated that plant-mediated AgNPs exhibit greater stability and lower cytotoxicity than chemically synthesized counterparts, making them promising candidates for biomedical applications [20, 21]. Additionally, AgNPs inhibit biofilm development by generating reactive oxygen species, which damage bacterial DNA and proteins, further compromising biofilm integrity [22, 23]. Furthermore, AgNPs enhance the stability, bioavailability, and targeted delivery of bioactive compounds, thereby reducing the required doses and minimizing potential side effects [24, 25]. Recent studies have demonstrated that AgNPs synthesized from *C. asiatica* and *Ayapana triplinervis* exhibit potent antimicrobial activity against wound pathogens, underscoring their potential as therapeutic agents for wound infections [26]. However, the synergistic effects of *C. asiatica*–derived bioactive compounds and AgNPs on oral biofilms remain underexplored.

Therefore, this study aims to explore the antibiofilm effects and biocompatibility of *C. asiatica* extract, its bioactive compounds (asiatic acid and madecassic acid), and their silver nanoparticle formulations against biofilm-forming *Streptococcus* species associated with oral diseases. By addressing the limitations of existing antimicrobial therapies, such as low efficacy, high cytotoxicity, and biofilm persistence, and leveraging the synergistic potential of nanotechnology and phytochemicals, this research contributes to the development of effective and biocompatible strategies for managing oral biofilms. The primary goal is to achieve an optimal balance between antimicrobial efficacy and cytotoxicity, thereby advancing the development of innovative therapeutic approaches to combat oral biofilm-related infections.

## 2. Materials and Methods

### 2.1. Ethical Approval

All the experiments were performed under the regulation of biosafety for scientific experiments (Ref. No. WU-IBC-65-007) of Walailak University, Nakhon Si Thammarat, Thailand.

### 2.2. Preparation of *C. asiatica* Ethanolic Extract

The preparation of *C. asiatica* ethanolic extract was conducted following the protocol described by Chonsut et al. [12]. Fresh *C. asiatica* leaves were obtained from a local supplier in Nakhon Si Thammarat Province, Thailand, washed thoroughly with running water, and air-dried at 37°C to preserve bioactive compounds. The dried leaves were ground into a fine powder using a mechanical grinder. The powdered leaves were then macerated in 95% ethanol at a plant-to-solvent ratio of 1:10 (w/v) for 72 h with occasional stirring. The extract was filtered through Whatman No. 1 filter paper to remove plant debris, and the filtrate was concentrated using a rotary evaporator under reduced pressure at 40°C. The resulting concentrate was freeze-dried to obtain a powder, which was stored at −20°C until further use. The extract yield was calculated as a percentage of the starting material. The freeze-dried extract was stored in amber vials to prevent photodegradation of bioactive compounds.

### 2.3. Green Synthesis of Silver Nanoparticles (AgNPs)

The green synthesis of AgNPs was performed as previously described [27]. Briefly, 1 mM solution of AgNO_3_ was prepared in deionized water. The *C. asiatica* ethanolic extract (10 mg/mL) was added dropwise to the AgNO_3_ solution in a 1:9 ratio (v/v) under continuous stirring at 60°C for 2 h. The reaction's pH was adjusted to 8 using 1 mM NaOH to optimize the reduction process. Formation of AgNPs was monitored by observing a color change to yellow-brown, indicating the surface plasmon resonance (SPR) of silver. UV-Vis spectroscopy (Perkin Elmer LAMBDA 25 UV/Vis spectrophotometer, Waltham, MA, USA) was performed to confirm peak absorption between 420 and 450 nm [28]. Scanning electron microscopy (SEM) was used to assess the particle size distribution and morphology. The synthesized nanoparticles were stored at 4°C for further experiments.

### 2.4. Bacterial Isolates and Culture Conditions

Standard strains of *S. mutans* ATCC 25175, *S. mitis* ATCC 49456, and *S. salivarius* ATCC 9759 were used in this study. Clinical isolates of biofilm-forming *S. mutans* were obtained from a previous study [29]. The bacterial isolates were cultured in brain heart infusion (BHI) broth supplemented with 2% sucrose to enhance biofilm formation. The cultures were incubated at 37°C in a 5% CO_2_ atmosphere for optimal growth. The bacterial suspension was diluted with 0.85% normal saline solution (NSS) to achieve an optical density (OD) of 0.1 (approximately 1 × 10^8^ CFU/mL) at 600 nm for antibacterial testing.

### 2.5. Antibacterial Activity Testing

The minimum inhibitory concentration (MIC) and minimum bactericidal concentration (MBC) of the crude *C. asiatica* ethanolic extract, the synthesized AgNPs, asiatic acid, and madecassic acid were determined using the broth microdilution method outlined by the Clinical and Laboratory Standards Institute [30]. Vancomycin was used as the positive control, while MHB medium, a 1% dimethyl sulfoxide (DMSO) solution, and blank AgNP served as negative controls. The bacterial suspensions (10^6^ CFU/mL) were added to wells containing serially diluted test agents and incubated at 37°C for 24 h. MIC was defined as the lowest concentration that showed no visible growth, while MBC was the lowest concentration that reduced bacterial viability by ≥ 99.9%, confirmed by subculturing on tryptic soy agar and observing bacterial growth after incubation at 37°C for 24 h. All determinations were performed in triplicate.

### 2.6. Inhibition of Bacterial Biofilm Formation Assay

The inhibitory effect of the test agents on bacterial biofilm formation was assessed by pretreating bacterial cells with sub-MIC concentrations (1/2 × MIC, 1/4 × MIC, and 1/8 × MIC) of the test agents for 4 h at 37°C. Following treatment, the cells were washed and diluted to an OD of 0.1 at 600 nm in fresh BHI-sucrose medium. Aliquots of 200 µL were added to sterile round-bottom 96-well polystyrene microplates (SPL Life Sciences Co., Korea) and incubated at 37°C for 24 h to promote biofilm development. The extent of biofilm formation was quantified using the crystal violet staining method as previously described by Karaolis et al. [31]. After incubation, the wells were rinsed with phosphate-buffered saline (PBS, pH 7.3), fixed with 99% methanol, and stained with 0.1% crystal violet for 15 min. The excess stain was removed, and the bound crystal violet was solubilized in 95% ethanol. The absorbance was measured at 590 nm using a microplate reader (BioTek Instruments, Inc., USA). The blank AgNPs and 0.2% chlorhexidine were used as the negative and positive controls, respectively. All experiments were performed in triplicate. The percentage inhibition of biofilm formation was calculated by comparing the OD values of treated and untreated samples using the formula: Percentage inhibition = (OD control − OD treatment/OD control) × 100.

### 2.7. Evaluation of the Effects of Test Agents on Bacterial Biofilm Reduction

To assess the reduction effect of the test agents on preformed biofilms, biofilms were initially grown under standard conditions. Following maturation, the medium was removed, and the wells were gently rinsed twice with PBS to eliminate nonadherent cells. Fresh BHI-sucrose medium (180 µL) was then added, followed by 20 µL of the test agents at a concentration of 1/2 × MIC. The treated biofilms were incubated, and the biofilm biomass was quantified at specific time intervals (0.5, 1, 4, 8, 12, and 24 h) using the crystal violet staining method. Absorbance measurements and percentage reductions were calculated as described previously.

### 2.8. SEM

The biofilms of *Streptococcus* sp. were prepared using a previously described method with slight modifications [32]. Briefly, biofilms were formed on 5-mm-diameter round glass coverslips placed in a flat-bottom 24-well polystyrene plate. The tested agents, including the *C. asiatica* ethanolic extract, asiatic acid, madecassic acid, and synthesized AgNPs, were added to the wells at their respective MICs. A 0.2% chlorhexidine solution was used as a positive control, while the medium containing 1% DMSO served as a negative control.

To grow mono-species biofilms, a culture medium containing 2% sucrose (1 mL) and bacterial cells (10^8^ CFU/mL) was anaerobically incubated at 37°C for 24 h. After biofilm formation, the coverslips were transferred to new wells containing 1 mL of 2.5% glutaraldehyde solution and fixed overnight. The fixed specimens were gently washed twice with PBS and then treated with 1% osmium tetroxide (OsO_4_) for 1 h. The biofilm samples were sequentially dehydrated in an ethanol concentration series (50, 70, 85, 90, 95, and 2 × 100%) for 15 min in each solution. The dehydrated biofilms were subsequently dried in a desiccator and coated with gold using a Cressington 108 Auto Sputter Coater (Cressington Scientific Instruments, UK). Each specimen was examined using a SEM (Merlin Compact, Zeiss, Germany) at the Center for Scientific and Technological Equipment (CSE), Walailak University. Surface topographies of the biofilm specimens were visualized in secondary electron emission mode at an accelerating voltage of 5 kV. The percentage of the biofilm-covered area on the coverslip surfaces was quantified using ImageJ software. Otsu's method was applied as a thresholding technique to distinguish biofilm from the background, ensuring clear biofilm visibility while minimizing background noise. A consistent region of interest (ROI) was defined, focusing on areas with visible biofilm. Multiple representative fields were included, selecting regions that exhibited distinct biofilm characteristics such as clustering, attachment, and layering for morphological analysis. Nonbiofilm regions were excluded from the study.

### 2.9. Cell Culture and Cytotoxicity Testing by MTT Assay

Cytotoxicity assessment was conducted to evaluate the biocompatibility of the synthesized AgNPs for potential therapeutic applications. Cytotoxicity of the test agents was evaluated using human gingival fibroblast cells (HGF-1) as described by Ajami et al. [33]. The dental fibroblast cells were cultured in Dulbecco's modified Eagle medium (DMEM) supplemented with 10% fetal bovine serum and 1% penicillin–streptomycin and incubated at 37°C in a 5% CO_2_ atmosphere. The cell suspension was seeded into 96-well plates at a density of 1 × 10^4^ cells/well and treated with the test agents at varying concentrations for 24 h. After treatment, MTT reagent (0.5 mg/mL) was added to each well, and the plates were incubated for 4 h. Formazan crystals formed by viable cells were dissolved in DMSO, and absorbance was measured at 570 nm. The cell viability assay was conducted in triplicate.

### 2.10. Statistical Analysis

All data were expressed as the mean ± standard error (SE) of the mean) and analyzed using one-way analysis of variance (ANOVA). For post hoc comparisons, Dunnett's multiple range tests were applied to identify significant differences between treatment groups and controls. Statistical significance was set at *p* < 0.05. Analyses were performed using SPSS software (IBM SPSS Statistics, version 26). This modified approach ensures compatibility with the study objectives, focusing on the antibiofilm effects of *C. asiatica*–derived agents and their nanotechnology-enhanced formulations.

## 3. Results

### 3.1. MIC and MBC Against *Streptococcus* sp.

The MIC and MBC results (Table 1) revealed the antimicrobial efficacy of tested agents against *Streptococcus* isolates. The synthesized AgNPs exhibited the highest potency, with MIC/MBC values of 62.5/125 µg/mL for *S. mutans* ATCC 25175 and *S. mitis* ATCC 49456. In contrast, the ethanolic extract of *C. asiatica* showed moderate antimicrobial activity, with MIC and MBC values ranging from 625 to 2500 µg/mL, indicating relatively lower activity compared to the synthesized AgNPs. The enhanced potency of asiatic acid and madecassic acid, with MIC values as low as 62.5 µg/mL for asiatic acid against *S. salivarius* and *S. mitis*. The MIC values of asiatic acid were approximately 10 times lower than the crude extract, which highlights the impact of bioactive compound purification on antimicrobial effectiveness. Vancomycin, the positive control, consistently exhibited strong activity (MIC/MBC: 0.5–1.0 µg/mL). The blank AgNPs (nonfunctionalized AgNPs) were inactive, emphasizing the critical role of *C. asiatica* phytochemicals in functionalized AgNPs, which significantly enhanced antimicrobial properties.

### 3.2. Biofilm Inhibition Activity

The biofilm inhibition activity of the tested agents varied significantly across *Streptococcus* species and concentrations (Table 2). The synthesized AgNPs demonstrated remarkably high biofilm inhibition efficiency, achieving over 75% inhibition at 1/2 × MIC for all tested strains. Notably, *S. mutans* isolate No. 5 was the most susceptible (77.53%), followed by *S. salivarius* (75.17%) and *S. mitis* (71.57%). This superior inhibition is indicative of the synergistic interaction between AgNPs and *C. asiatica* bioactives, which disrupts biofilm formation and integrity. At lower concentrations (1/8 × MIC and 1/4 × MIC), the synthesized AgNPs retained moderate antibiofilm activity (35.10%–63.90%), suggesting that even subinhibitory concentrations interfere with initial adhesion and EPS formation. The extract and triterpenoids displayed a similar dose-dependent pattern but required higher concentrations for comparable activity.

In comparison, the ethanolic extract of *C. asiatica* demonstrated moderate inhibition (16.07%–55.60%), with the highest rates at 1/2 × MIC against *S. salivarius* ATCC 9759 (53.57%) and *S. mutans* ATCC 25175 (55.60%). Clinical isolate No. 31 of *S. mutans* was the least affected (41.77%). Despite its lower potency compared to the synthesized AgNPs, the extract exhibited a clear concentration-dependent trend, with significant improvement at higher concentrations.

Asiatic acid and madecassic acid exhibited lower efficacy than both the extract and the synthesized AgNPs. The AgNP control (AgNPs without *C. asiatica* phytochemicals) exhibited minimal activity (< 18.57% for all strains), emphasizing the role of *C. asiatica* phytochemicals in enhancing biofilm disruption. The 0.2% chlorhexidine, as a positive control, consistently demonstrated superior biofilm inhibition (77.33%–83.30%), affirming its standard antimicrobial potency.

### 3.3. Time-Kinetics of Biofilm Reduction

The time-dependent biofilm reduction at 1/2 × MIC is summarized in Table 3. The synthesized AgNPs exhibited rapid and sustained antibiofilm activity, achieving a 76% reduction within 12 h and 79% at 24 h, significantly outperforming the *C. asiatica* extract, which achieved 54% and 57% biofilm reduction at the same time points. At the initial time point (0.5 h), the synthesized AgNPs demonstrated 21% biofilm reduction, slightly exceeding the extract (19%). By 1 h, biofilm biomass was reduced by 50% with the synthesized AgNPs, compared to 35% with the extract. The gap widened at 4 h, where the synthesized AgNPs achieved a 64% reduction, while the extract reached 39%. Notably, the synthesized AgNPs demonstrated more rapid biofilm reduction at earlier time points, indicating their potential to disrupt biofilm integrity even at subinhibitory concentrations. The AgNP control, lacking phytochemical functionalization, exhibited minimal activity (15%–25% reduction), emphasizing the pivotal role of *C. asiatica* bioactives in enhancing AgNP efficacy. In contrast, 0.2% chlorhexidine, used as a positive control, demonstrated the most potent antibiofilm activity, achieving a 90% reduction at 24 h. Its rapid and broad-spectrum effects reaffirm its effectiveness as a standard antimicrobial agent.

The superior performance of synthesized AgNPs compared to the crude extract highlights the synergistic interaction between AgNPs and *C. asiatica* bioactives, reinforcing their potential as a novel antibiofilm strategy.

### 3.4. Morphological Observation of *Streptococcus* Biofilms by SEM

**Figure 1:**
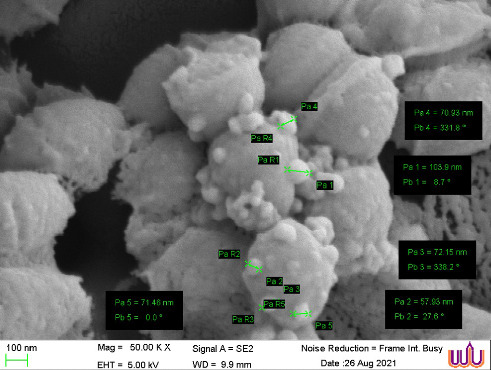
Scanning electron microscopy (SEM) images captured at × 50,000 magnification, illustrating the size and morphological characteristics of silver nanoparticles (AgNPs) synthesized from *C. asiatica* ethanolic extract. The AgNPs exhibit a spherical shape, uniform distribution, and an average size of 50–100 nm, highlighting their nanoscale structure and potential for antibiofilm applications.

SEM analysis provided critical insights into the morphology and distribution of the synthesized AgNPs functionalized with *C. asiatica* ethanolic extract. The nanoparticles exhibited a spherical shape with uniform dispersion, measuring approximately 50–100 nm in diameter, indicative of their structural stability and suitability for antibiofilm applications (Figure 1).

**Figure 2:**
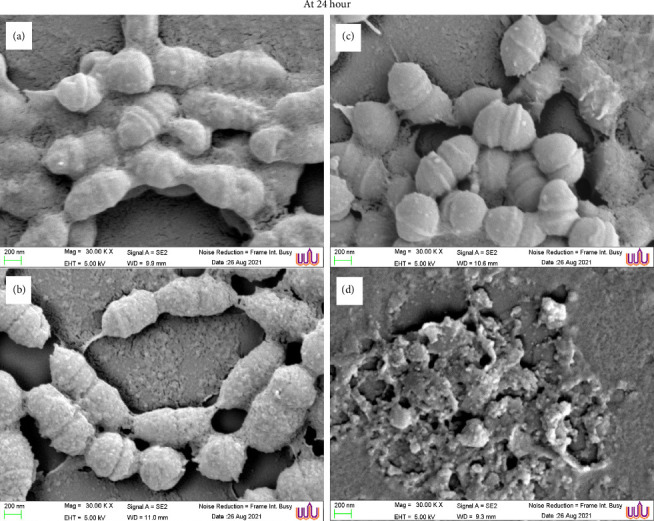
Scanning electron microscopy (SEM) images comparing the biofilm characteristics of *S. mutans* ATCC 25175 after 24 h of treatment. Images were captured at × 30,000 magnification, showing the untreated control (a), treatment with 1/2 × MIC of *C. asiatica* ethanolic extract (b), 1/2 × MIC of synthesized AgNPs (c), and 0.2% chlorhexidine (d). The AgNP control is included for reference.

Furthermore, SEM imaging of *S. mutans* biofilms treated with different agents revealed distinct morphological changes (Figure 2). Untreated biofilms (negative control) exhibited a dense, well-structured matrix with abundant EPS and tightly packed bacterial clusters (Figure 2(a)). In contrast, biofilms exposed to 1/2 × MIC of the synthesized AgNPs exhibited substantial structural disruption, characterized by extensive EPS degradation. While the bacterial cells exhibited slight contraction compared to the control, they remained intact without signs of disintegration. This corresponded to a 79% reduction in biomass at 24 h (Table 3), which was primarily attributed to the degradation of EPS rather than bacterial cell loss (Figure 2(c)). Treatment with 1/2 × MIC of *C. asiatica* ethanolic extract resulted in moderate biofilm disruption, with noticeable thinning of the EPS layers and partial disaggregation of bacterial clusters. However, bacterial cell morphology remained similar to that of the control, with no evidence of cell disintegration (Figure 2(b)). The most severe biofilm degradation was observed in biofilms treated with 0.2% chlorhexidine, which exhibited minimal EPS, extensively deformed bacterial cells, and near-complete biofilm eradication (Figure 2(d)). This corresponded to a 90% reduction in biomass (Table 3), attributed to both bacterial destruction and biofilm disintegration.

These findings highlight the superior antibiofilm potential of the synthesized AgNPs compared to the crude extract, demonstrating their ability to effectively disrupt biofilm matrix integrity while preserving bacterial cell morphology, suggesting a targeted antibiofilm strategy rather than indiscriminate bactericidal action.

### 3.5. Cytotoxicity Assessment

The cytotoxic effects of *C. asiatica* ethanolic extract, the synthesized AgNPs, asiatic acid, madecassic acid, the AgNP control, and 0.2% chlorhexidine were evaluated on HGF-1 cells at 1/2 × MIC using the MTT assay (Table 4). The synthesized AgNPs exhibited moderate cytotoxicity with 68.3% cell viability, whereas the AgNP control maintained a higher viability (82.4%), suggesting that phytochemical functionalization with *C. asiatica* enhanced antimicrobial potency but slightly increased cytotoxicity. The ethanolic extract and its bioactive components (asiatic acid and madecassic acid) showed acceptable biocompatibility, with asiatic acid demonstrating the highest cell viability (88.6%). In contrast, 0.2% chlorhexidine displayed the lowest cell viability (55.2%), aligning with its well-documented cytotoxic effects at therapeutic concentrations. However, its clinical application typically involves short exposure durations, mitigating prolonged cytotoxic risks.

The results underscore the balanced efficacy and biocompatibility of the synthesized AgNPs, making them promising candidates for therapeutic applications where both antimicrobial performance and safety are crucial.

## 4. Discussion

Biofilm-producing *Streptococcus* species, including certain strains of *S. mutans*, play a critical role in bacterial survival and pathogenicity within the oral cavity. By facilitating colonization and adherence to the enamel surface, these biofilms contribute to plaque accumulation and tooth damage [2]. Inhibiting biofilm formation is essential for preventing dental caries. However, the increasing prevalence of multidrug-resistant *S. mutans* raises concerns regarding the long-term efficacy of conventional antibiotics and chemical agents used in dentistry [7]. Consequently, plant-derived compounds have emerged as promising alternatives due to their broad-spectrum antimicrobial activity and lower risk of resistance development.

*C. asiatica* has been historically recognized for its medicinal properties across various cultures and traditional medical systems. Previous studies have demonstrated that ethanolic extracts of *C. asiatica* exhibit antimicrobial activity against *S. mutans*, with MIC values exceeding 1000 µg/mL [12]. This aligns with the findings of the present study, which determined the MIC of *C. asiatica* extract to be 625-1250 µg/mL against both standard and clinical *Streptococcus* isolates. These results indicate that *C. asiatica* extract exhibits moderate antibacterial activity against cariogenic bacteria.

Nanotechnology offers innovative approaches to enhance the efficacy of natural antimicrobial agents. AgNPs synthesized using plant extracts have demonstrated significant antibiofilm activity [18]. Unlike conventional antimicrobials, AgNPs can penetrate the EPS matrix, destabilize microbial membranes, and disrupt metabolic pathways, thereby increasing bacterial susceptibility [19]. Previous studies have also reported that plant-mediated AgNPs exhibit greater stability and lower cytotoxicity than their chemically synthesized counterparts, making them promising candidates for biomedical applications [20, 21].

This study provides compelling evidence of the potent antibiofilm activity of AgNPs synthesized from *C. asiatica* extract against oral *Streptococcus* pathogens. The results highlight the crucial role of *C. asiatica* phytochemicals, particularly asiatic acid and madecassic acid, in enhancing AgNP bioactivity. The MIC values of the synthesized AgNPs were approximately ten times lower than those of the crude extract, suggesting a synergistic effect between AgNPs and *C. asiatica* bioactive compounds. UV-Vis spectroscopic analysis revealed a distinct SPR peak at approximately 420–450 nm, indicative of AgNP formation and consistent with previous findings. The SPR phenomenon results from the collective oscillation of conduction electrons at the nanoparticle surface in response to incident light. The position and intensity of this peak depend on nanoparticle size, shape, and the surrounding dielectric environment. Additionally, the negative zeta potential (−32 mV) of the synthesized AgNPs indicates high colloidal stability, preventing aggregation and ensuring uniform dispersion. The SPR properties of AgNPs are also closely linked to their antimicrobial activity [18].

Previous research has demonstrated that AgNPs synthesized using *C. asiatica* extracts exhibit enhanced bioactivity against major wound pathogens, with inhibition zones ranging from 15 to 19 mm, along with antioxidant and anti-inflammatory properties [26]. Moreover, AgNPs derived from *C. asiatica* leaf extract have shown strong antimicrobial activity against *Staphylococcus aureus* and *Pseudomonas aeruginosa*, with inhibition zones of 22.5 mm and 25 mm, respectively [34].

Our findings indicate that the synthesized AgNPs achieved 76% biofilm biomass reduction within 12 h and sustained activity up to 79% at 24 h, outperforming the moderate effects of crude *C. asiatica* extract, which reduced biofilm biomass reduction by only 57%. This suggests that phytochemical-functionalized AgNPs significantly enhance antibiofilm activity beyond that of the crude extract alone. These findings align with previous reports that green-synthesized AgNPs exhibit superior biofilm inhibition compared to unfunctionalized AgNPs and crude plant extracts due to their unique physicochemical properties, including a high surface-area-to-volume ratio, nanoscale size, and controlled Ag^+^ ion release [35–37]. Other studies have demonstrated that plant-derived compounds such as quercetin and flavonoids enhance AgNP antibacterial properties by disrupting bacterial membranes, inhibiting quorum sensing, and preventing EPS formation [38].

Furthermore, SEM imaging of *S. mutans* biofilms treated with different agents revealed distinct morphological alterations. Biofilms exposed to 1/2 × MIC of the synthesized AgNPs exhibited substantial structural disruption, characterized by extensive EPS degradation. While the bacterial cells showed slight contraction compared to the untreated control, they remained structurally intact without evidence of lysis. This corresponded to a 79% reduction in biomass at 24 h, primarily due to EPS degradation rather than bacterial cell loss.

EPS is a key structural component of bacterial biofilms and plays a crucial role in the pathogenicity of *S. mutans*, the primary cariogenic bacterium in the oral cavity. EPS facilitates bacterial adhesion, promotes biofilm stability, and enhances resistance to host immune responses and antimicrobial agents, contributing to the persistence of dental plaque and the progression of dental caries [39]. Among biofilm-producing *Streptococcus* species, EPS production is considered a major virulence determinant that supports bacterial survival and pathogenicity. The synthesis of EPS in *S. mutans* is primarily regulated by glucosyltransferases (Gtfs), key enzymes involved in quorum sensing and biofilm formation. *S. mutans* encodes three types of Gtfs, including GtfC, which is essential for producing both soluble and insoluble glucans, thereby facilitating bacterial aggregation and adherence to the enamel surface [40]. Previous studies have demonstrated that molecular docking analysis of natural compounds derived from *C. asiatica*, such as asiatic acid, madecassic acid, and madasiatic acid, revealed interactions with multiple amino acid residues in the catalytic site of GtfC. These interactions, mediated by hydrogen bonding and hydrophobic forces, may hinder the utilization of sucrose as a substrate for water-insoluble glucan synthesis [12]. Furthermore, madecassic acid has been shown to reduce bacterial adhesion by disrupting hydrophobic interactions and inhibiting quorum sensing, both of which are essential for biofilm maturation [13, 16].

The nanoscale properties of AgNPs further enhance their antibiofilm efficacy. AgNPs can penetrate deep into the EPS matrix, destabilizing its structure and increasing bacterial susceptibility [19]. Additionally, Ag^+^ ions bind to bacterial DNA and ribosomes, inhibiting transcription and translation, thereby exerting bacteriostatic effects [41]. SEM imaging in this study revealed substantial biofilm disruption following AgNP treatment, reinforcing the role of SPR-mediated electron oscillations in antimicrobial activity. These findings align with previous research linking the SPR effect to AgNP functionality [14]. This study provides further evidence that incorporating bioactive compounds from *C. asiatica* into AgNPs significantly enhances their antibiofilm effects, increasing bacterial susceptibility and promoting biofilm degradation. Therefore, a potential mechanism by which AgNPs synthesized from *C. asiatica* extract exert antimicrobial activity against oral *S. mutans* may involve EPS disruption without inducing bacterial cell lysis.

In comparison with 0.2% chlorhexidine, which achieved 90% biofilm inhibition at 24 h through membrane permeabilization and EPS inhibition, the synthesized AgNPs demonstrated comparable biofilm disruption while maintaining significantly higher biocompatibility. Chlorhexidine exhibited high cytotoxicity, reducing HGF-1 cell viability to 55.2%, which limits its prolonged use, as previously reported in studies on oral cytotoxicity [42, 43]. In contrast, the synthesized AgNPs, functionalized with *C. asiatica* phytochemicals, retained 68.3% cell viability at 1/2 × MIC, suggesting a favorable therapeutic window. Since the therapeutic window, the difference between the effective antimicrobial concentration and the cytotoxic concentration, is crucial for antimicrobial development [44], these findings support the suitability of AgNPs for therapeutic applications. However, since HGF-1 cells may not fully represent the cellular environment directly exposed to biofilms, future studies should incorporate oral epithelial cell lines (e.g., OKF6/TERT-2, TR146) to enhance the clinical relevance of toxicity assessments and ensure the safety of AgNP-based therapies for oral biofilm-associated infections [45].

## 5. Conclusions

This study highlights the potent antibiofilm activity of silver nanoparticles (*C. asiatica*–functionalized AgNPs) against *S. mutans* and other biofilm-forming *Streptococcus* species associated with oral diseases. Compared to the crude *C. asiatica* extract, the synthesized AgNPs exhibited superior biofilm reduction, achieving over 75% inhibition within 12 h and sustaining efficacy up to 79% at 24 h. This enhanced performance is attributed to the synergistic effects of AgNPs and *C. asiatica* bioactives, which promote EPS degradation without inducing cell lysis. Cytotoxicity assessments confirmed an optimal balance between antimicrobial potency and biocompatibility, with 68% cell viability at 1/2 × MIC, suggesting their suitability for therapeutic applications. These findings underscore the potential of *C. asiatica*–functionalized AgNPs as a nanotechnology-driven alternative for oral biofilm-associated infections. Future studies should explore molecular interactions, transcriptomic analysis of biofilm genes, toxicity assessments in oral epithelial cell lines, and *in vivo* efficacy to ensure clinical relevance and safety. This study provides a foundation for developing *C. asiatica* AgNPs as a sustainable antimicrobial agent for oral healthcare.

## Figures and Tables

**Table 1:** Minimum inhibitory concentrations (MICs) and minimum bactericidal concentrations (MBCs) of tested agents against *Streptococcus* species.

Bacterial strains	MIC/MBC of antibacterial agents (μg/mL)					
	Ethanolic extract of *C. asiatica*	Synthesized AgNPs	Asiatic acid	Madecassic acid	AgNPs control	Vancomycin
*S. mutans* ATCC 25175	1250/2500	62.5/125	125/500	250/500	NA	0.5/1.0
*S. mutans* clinical isolate no. 5	625/1250	62.5/125	250/500	500/1000	NA	0.5/1.0
*S. mutans* clinical isolate no. 15	1250/2500	125/250	250/500	250/500	NA	1.0/1.0
*S. mutans* clinical isolate no. 31	1250/2500	125/250	125/250	125/250	NA	0.5/1.0
*S. mitis* ATCC 49456	625/2500	62.5/125	62.5/250	125/250	NA	0.5/1.0
*S. salivarius* ATCC 9759	625/1250	125/250	62.5/125	500/1000	NA	0.5/1.0

Abbreviation: NA, not active.

**Table 2:** Percentage of biofilm formation inhibition by tested agents against *Streptococcus* species.

Agent	Concentration	Biofilm inhibition (%)					
		*S. mutans* ATCC 25175	*S. mutans* clinical no. 5	*S. mutans* clinical no. 15	*S. mutans* clinical no. 31	*S. mitis* ATCC 49456	*S. salivarius* ATCC 9759
*C. asiatica* extract	1/8 × MIC	25.30 ± 1.2	22.23 ± 0.9	20.70 ± 1.8	16.07 ± 1.0	20.20 ± 1.2	23.97 ± 1.8
	1/4 × MIC	40.80 ± 1.8	35.27 ± 1.3	35.97 ± 1.2	34.37 ± 0.9	31.33 ± 1.0	36.43 ± 1.2
	1/2 × MIC	55.60 ± 1.0	45.17 ± 0.8	46.93 ± 1.0	41.77 ± 1.2	46.60 ± 1.4	53.57 ± 1.1

Synthesized AgNPs	1/8 × MIC	45.20 ± 1.4	46.27 ± 1.2	47.20 ± 1.8	39.63 ± 1.6	39.07 ± 1.4	35.10 ± 1.0
	1/4 × MIC	63.90 ± 0.9	59.50 ± 1.4	60.67 ± 1.2	57.70 ± 0.7	62.27 ± 1.8	63.33 ± 1.8
	1/2 × MIC	78.40 ± 1.2	77.53 ± 1.8	75.43 ± 0.9	76.03 ± 1.2	71.57 ± 0.9	75.17 ± 1.2

Asiatic acid	1/8 × MIC	18.40 ± 1.0	17.03 ± 1.1	16.30 ± 1.6	13.47 ± 1.8	17.83 ± 1.2	13.60 ± 0.8
	1/4 × MIC	32.80 ± 1.8	28.20 ± 0.9	32.47 ± 1.2	20.83 ± 1.0	24.70 ± 1.0	21.30 ± 0.9
	1/2 × MIC	47.10 ± 1.1	42.93 ± 1.0	39.07 ± 1.6	19.83 ± 0.9	33.03 ± 1.2	37.80 ± 1.4

Madecassic acid	1/8 × MIC	16.50 ± 0.9	15.23 ± 1.2	15.33 ± 0.7	17.00 ± 1.4	14.95 ± 1.1	14.57 ± 1.2
	1/4 × MIC	28.30 ± 1.2	24.77 ± 1.8	25.80 ± 1.0	19.83 ± 1.2	29.27 ± 1.8	27.03 ± 1.0
	1/2 × MIC	39.70 ± 1.8	35.37 ± 1.0	30.30 ± 1.8	20.33 ± 0.9	35.20 ± 1.2	36.03 ± 1.0

AgNPs control	—	18.57 ± 0.9	17.10 ± 1.4	15.74 ± 1.2	17.06 ± 1.4	17.78 ± 1.1	17.63 ± 0.9

0.2% chlorhexidine	—	82.03 ± 1.2	78.97 ± 1.8	77.99 ± 0.9	77.33 ± 1.6	81.03 ± 1.8	83.30 ± 1.2

*Note:* Data are presented as the mean ± SE from three independent experiments.

**Table 3:** Percentage of biofilm biomass reduction of *S. mutans* ATCC 25175 over 24 h after treatment with *C. asiatica* ethanolic extract and synthesized AgNPs at the concentration of 1/2 × MIC.

Time (h)	Biofilm biomass reduction (%)			
	*C. asiatica* extract	Synthesized AgNPs	AgNP control	0.2% chlorhexidine
0.5	19 ± 2.1	21 ± 3.0	15 ± 2.5	48 ± 2.8
1	35 ± 3.5	50 ± 3.8	19 ± 3.2	69 ± 3.7
4	39 ± 2.1	64 ± 4.2	21 ± 2.8	80 ± 3.0
8	55 ± 3.5	70 ± 1.9	25 ± 2.9	82 ± 3.1
12	54 ± 2.9	76 ± 2.4	19 ± 1.2	84 ± 4.5
24	57 ± 1.3	79 ± 3.5	22 ± 3.9	90 ± 2.3

*Note:* Data are presented as the mean ± SE from three independent experiments.

**Table 4:** Cell viability of human gingival fibroblast cells (HGF-1) treated with test agents at the concentration of 1/2 × MIC using MTT assay.

Test agent	Cell viability (%)
*C. asiatica* ethanolic extract	75.4 ± 2.1
Synthesized AgNPs	68.3 ± 1.8
Asiatic acid	88.6 ± 1.9
Madecassic acid	77.2 ± 2.3
AgNP control	82.4 ± 1.7
0.2% chlorhexidine	55.2 ± 1.5

*Note:* Data are presented as the mean ± SE from three independent experiments.

## Data Availability

The datasets generated during the current study are available from the corresponding author upon reasonable request.
